# Advancing Age-Friendly Care Through Lifestyle Medicine Education

**DOI:** 10.1093/geroni/igaf122.1356

**Published:** 2025-12-31

**Authors:** Kristen Neises

**Affiliations:** University of Louisville, Louisville, Kentucky, United States

## Abstract

The integration of age- and dementia-friendly principles into healthcare education is essential for preparing a workforce capable of delivering high-quality care to older adults. Traditional training often lacks a preventive, interdisciplinary approach that addresses the role of lifestyle factors in aging and cognitive health. Through my Geriatric Academic Career Award (GACA)-funded initiative, I have developed a comprehensive geriatrics curriculum that embeds lifestyle medicine into interprofessional education, ensuring that learners across disciplines gain the skills needed to support healthy aging and dementia prevention. This project incorporates case-based virtual learning, a micro-credentialing course in age- and dementia-friendly lifestyle medicine, and the infusion of lifestyle medicine principles into interprofessional education involving medicine, nursing, social work, paraprofessionals, pharmacy, and other disciplines. By fostering collaboration and strengthening workforce competency, this curriculum enhances learners’ knowledge, confidence, and ability to apply lifestyle-based interventions in older adult care. This presentation will highlight the impact of this educational approach, discuss challenges and opportunities in sustaining and scaling geriatrics training, and explore how embedding lifestyle medicine into workforce education can improve patient outcomes and better prepare healthcare professionals to address the needs of an aging population.

